# Generational Multimorbidity Disease Clusters for British Cohorts Born 1921 – 1960

**DOI:** 10.1093/geroni/igab046.3399

**Published:** 2021-12-17

**Authors:** Stacey Voll, Graciela Muniz-Terrera, Scott Hofer

**Affiliations:** 1 University of Victoria, Victoria, British Columbia, Canada; 2 University of Edinburgh, Edinburgh, Scotland, United Kingdom

## Abstract

The aim of this study is the first step in our understanding of the uniqueness and stability of multmorbdity disease patterns for different generations. The unique historical context that each generation has been exposed to is thought to have systemic health impacts and differences in epidemiological make-up (Clouston et al. 2021). Literature suggests that multimorbidity disease patterns, are similar across countries (Hernandez et al, 2021 – in press) and observational points, and that migration into complex disease clusters is more common as people age (Cassell et al, 2018, Kingston et al. 2018). Most commonly reported are Cardiovascular and Metabolic disease clusters which lead to lower quality of life, mortality and morbidity (Kudesia, 2021). We asked: Do multimorbidity disease patterns differ for unique generations? Using the ELSA, the disease clusters of three cohorts were examined; an older cohort, born 1921-1930, a middle cohort born 1931-1940 a younger cohort born 1941-1950 and the ”newest” cohort, born 1951-1960. Self-reported dementia and memory problems lead a specific cluster for the middle cohort, those born in 1931-1940, but not for the other cohorts. While disease patterns were different between sex for other clusters, the disease cluster of dementia and memory problems held similar disease patterns for males and females, with a prevalence of 3%. The dementia/memory problem cluster loaded with cardio/metabolic diseases. This suggests that complex multimorbidity for the British 1931-1940 cohort has had an impact related to dementia and memory problem diagnoses for this specific generation, for males and females alike.

